# Mapping educational opportunities for healthcare workers on antimicrobial resistance and stewardship around the world

**DOI:** 10.1186/s12960-018-0270-3

**Published:** 2018-02-05

**Authors:** Susan Rogers Van Katwyk, Sara L. Jones, Steven J. Hoffman

**Affiliations:** 1 Global Strategy Lab, Dahdaleh Institute for Global Health Research, Faculty of Health and Osgoode Hall Law School, York University, 4700 Keele Street, Toronto, ON M3J 1P3 Canada; 2 School of Epidemiology and Public Health, University of Ottawa, Ottawa, ON Canada; 3 Department of Health Research Methods, Evidence, and Impact and McMaster Health Forum, McMaster University, Hamilton, ON Canada; 4 Department of Global Health and Population, Harvard T.H. Chan School of Public Health, Harvard University, Boston, MA USA; 5 Dalhousie Medical School, Dalhousie University, Halifax, NS Canada

**Keywords:** AMR, Antimicrobial resistance, Antimicrobial stewardship, Education, Healthcare workers

## Abstract

**Background:**

Antimicrobial resistance is an important global issue facing society. Healthcare workers need to be engaged in solving this problem, as advocates for rational antimicrobial use, stewards of sustainable effectiveness, and educators of their patients. To fulfill this role, healthcare workers need access to training and educational resources on antimicrobial resistance.

**Methods:**

To better understand the resources available to healthcare workers, we undertook a global environmental scan of educational programs and resources targeting healthcare workers on the topic of antimicrobial resistance and antimicrobial stewardship. Programs were identified through contact with key experts, web searching, and academic literature searching. We summarized programs in tabular form, including participating organizations, region, and intended audience. We developed a coding system to classify programs by program type and participating organization type, assigning multiple codes as necessary and creating summary charts for program types, organization types, and intended audience to illustrate the breadth of available resources.

**Results:**

We identified 94 educational initiatives related to antimicrobial resistance and antimicrobial stewardship, which represent a diverse array of programs including courses, workshops, conferences, guidelines, public outreach materials, and online-resource websites. These resources were developed by a combination of government bodies, professional societies, universities, non-profit and community organizations, hospitals and healthcare centers, and insurance companies and industry. Most programs either targeted healthcare workers collectively or specifically targeted physicians. A smaller number of programs were aimed at other healthcare worker groups including pharmacists, nurses, midwives, and healthcare students.

**Conclusions:**

Our environmental scan shows that there are many organizations working to develop and share educational resources for healthcare workers on antimicrobial resistance and antimicrobial stewardship. Governments, hospitals, and professional societies appear to be driving action on this front, sometimes working with other types of organizations. A broad range of resources have been made freely available; however, we have noted several opportunities for action, including increased engagement with students, improvements to pre-service education, recognition of antimicrobial resistance courses as continuing medical education, and better platforms for resource-sharing online.

## Background

Antimicrobial resistance (AMR) is a looming global health problem; misuse and overuse of antimicrobials in the past 50 years has accelerated the development of resistance to antibiotics and other antimicrobial drugs [1]. With few new antibiotics in development [2–4], it is essential that we preserve the usefulness of existing antimicrobials. In their capacity as prescribers and educators, healthcare workers (HCWs) will need to shoulder much of the burden in stewarding the preservation and longevity of antimicrobial agents. The inappropriate use of antimicrobials—such as prescribing antibiotics for viral illnesses or prescribing broad spectrum antibiotics where a narrow spectrum antibiotic would be preferable [5]—drives the development of resistance [6]. In making smart prescribing choices, HCWs can reduce genetic selection pressures that drive the development of resistance [1]. Beyond prescribing, HCWs have roles in infection prevention and control (IPC), in advocating for rational antibiotic use, and in educating their patients on the responsible use of antibiotics.

Changing HCW and patient habits will require substantial effort to ensure that all prescribers and patients are aware of the challenges associated with AMR. Surveys suggest that many HCWs receive too little training on AMR and rational antibiotic use. In Saudi Arabia, nearly half of surveyed physicians felt that they had inadequate knowledge of antibiotic prescribing [7]. The Infectious Disease Society of America has previously stated that clinician training on antimicrobial use in the United States of America (USA) is highly variable and non-standardized [8], and medical students in both Europe and the USA have reported a desire for increased training on choosing antibiotic therapies [9, 10]. The lack of coordinated approaches to AMR education of HCWs poses a challenge to ensuring prudent antimicrobial prescribing.

The WHO Global Action Plan on Antimicrobial Resistance expresses a need for all countries to include antibiotic resistance as a core component of both HCW training and continuing education [11]. To better understand this field, we present an illustrative mapping of educational programs and resources on AMR and antimicrobial stewardship (AMS) aimed at HCWs. This effort included an investigation of resources for AMR/AMS education, opportunities for continuing education, and actors in the field. This mapping exercise has allowed us to understand existing efforts and identify key areas for action in AMR education.

## Methods

We sought to identify AMR educational programs targeting HCWs. For breadth, programs were identified through multiple methods, including contact with key experts, web searching, and academic literature searching. An initial literature search in August and September 2016 identified educational programs and key academic researchers who had published in the field over the previous decade. We emailed these and other key experts identified by WHO regional office contacts to request information on AMR and AMS-related educational programs. Additional experts were identified via a snowball strategy and web-searching. Experts were asked to identify recent or ongoing educational programs on AMR/AMS.

We further identified educational programs through online web-searching. We hand-searched the websites of several relevant organizations to identify past and current educational initiatives. We also conducted a non-systematic web search using combinations of a range of search-terms including “antimicrobial resistance”, “antibiotic resistance”, “antimicrobial stewardship,” “antibiotic stewardship”, “education”, “course”, “curriculum”, and “workshop” and region or country in September and October 2016. Relevant articles, project reports, course flyers, and other resources were reviewed to identify programs. We examined national action plans concerning AMR in search of references to educational initiatives. Once identified, these programs were followed up with additional online research as necessary.

We summarized the identified programs in tabular form, including participating organizations, program types, region, and intended audience. We created codes to classify programs by program type, organization type, and WHO region. Programs that were not associated with any specific region (such as resources that were globally available and without a geographical target population) were classified as “global”. Multiple program codes were assigned as necessary. Program type and organization codes were developed and updated iteratively as programs were identified to identify commonalities between initiatives.

## Results

### Mapping educational initiatives

In total, we identified 94 educational initiatives related to AMR/AMS. The 94 initiatives represent a diverse array of programs including formal courses, workshops, conferences, guidelines, public outreach materials, and online-resource websites. The complete list of initiatives is presented in Table 1.

**Table 1:** Listing of identified educational initiatives

#	Program	Lead organization	Country	Region	Target audience	Description	
1	Clinical Antibiotic Stewardship for South Africa	South African Antibiotic Stewardship Programme	South Africa	Africa	Medical students, physicians	This self-paced online course aims to introduce antimicrobial stewardship, describe how to implement stewardship programs, provide guidance for prescribing and interpreting clinical results, and teach about infection prevention and control. The course is taught online through video lectures and is free. *https://www.openlearning.com/courses/clinical-antibiotic-stewarship-for-south-africa*	^a,b^
2	Distance Learning Course in Antimicrobial Stewardship for Africa	Infection Control Africa Network	Multiple	Africa	Physicians, clinical pharmacists, clinical microbiologists, nurses	This 3.5-month-long online distance learning course comprises five modules relating to infectious diseases, one of which is focused on Antimicrobial Stewardship. The program includes lectures, reading materials, and discussion forums to engage with tutors and other students. Program is free for African participants. *http://www.icanetwork.co.za/education/distance-learning-course-in-antimicrobial-stewardship-for-africa/*	^a,b^
3	Online Rational Medicines Use Module	University of the Western Cape	South Africa	Africa	Healthcare professionals	This semester-long online course focuses on the rational use of medicines and associated problems. Content is taught through online media and reading materials, and learning is encouraged through weekly tasks, online discussion forums, and written assignments. *http://www.fidssa.co.za/Content/Documents/UWC_RATIONAL_MEDICINES_USE_CE_MODULE_2015_ADVERTFinal_(2).pdf*	^b^
4	Antibiotic Pharmacology Webcasts	Cleveland Clinic: Centre for Continuing Education	USA	Americas	Physicians	A collection of 6 continuing education webcasts on AMR focusing on different types of antimicrobial. Webcasts can be completed for credit towards the American Medical Association’s Physician Recognition Award Credit System. *http://www.clevelandclinicmeded.com/online/pharmacology/pharm-antibiotic.htm*	^b^
5	Antimicrobial Management Team	University of Kentucky	USA	Americas	Healthcare professionals	Resource website including policies, institution-specific antibiotic guidelines, and personnel training resources. *http://www.hosp.uky.edu/pharmacy/amt/default.html*	
6	Antimicrobial Stewardship - Moving Knowledge to Action	Public Health Ontario	Canada	Americas	Healthcare professionals	A workshop series focused on providing support to develop and grow hospital AMR stewardship programs. Workshops included lectures, participant networking, and highlighted local experiences. The workshops occurred in 4 cities in Ontario and were broadcast via teleconference. Presentation notes and slides are available freely online. *https://www.publichealthontario.ca/en/BrowseByTopic/InfectiousDiseases/AntimicrobialStewardshipProgram/Pages/Antimicrobial_Stewardship_Workshop.aspx*	^a^
7	Antimicrobial Stewardship Program (ASP) at Nebraska Medicine	Nebraska Medicine	USA	Americas	Healthcare professionals	Program includes video lectures, restricted antimicrobial list, institution-specific antibiotic guidelines, microbiology information, and other educational material. A mobile app provides clinical guidelines, protocols, and dosing adjustments for antimicrobial use. Many web sources are available freely online, but the app requires specific login credentials. *http://www.nebraskamed.com/careers/education-programs/asp*	^a^
8	Antimicrobial Stewardship: A tipping point, transforming practice	Mount Sinai Hospital-University Health Network	Canada	Americas	Physicians, pharmacists	A three-day workshop to identify key ASP priorities and resources, better understand the microbiology laboratory, and consider infectious disease management from an ASP perspective. *Weblink no longer available; Courtesy of Andrew Morris Mount Sinai Hospital.*	
9	Antimicrobial Stewardship: Optimization of Antibiotic Practices	Stanford University	USA	Americas	Healthcare professionals	A MOOC covering the Infectious Disease Society of America guidelines, stewardship principles for special populations, and evidence based antibiotic management for surgical prophylaxis, outpatient care, and sepsis treatment. *http://www.euro.who.int/en/health-topics/disease-prevention/antimicrobial-resistance/news/news/2014/05/massive-online-open-course-mooc-on-antimicrobial-stewardship*	
10	Antimicrobial Stewardship: Taking it to the Next Level	Mount Sinai Hospital-University Health Network	Canada	Americas	Physicians, pharmacists	Offered in 2011, a course providing information on how to (1) prepare a business case for funding an ASP, (2) identifying the data elements required to start and monitor an ASP, (3) choose ASP priorities, (4) change prescriber behavior, (5) report success and challenges. Continuing Education credit was available for this course. *http://www.antimicrobialstewardship.com/educational-opportunities*	^b^
11	Bugs and Drugs	Alberta Health Services	Canada	Americas	Healthcare professionals	The Bugs & Drugs reference guide provides recommendations for antimicrobial use and infection prevention and management to improve patient care and limit AMR, toxicity, and costs. It is available as a book and mobile app. *http://www.bugsanddrugs.ca/thebook.php*	^a^
12	Cleveland Clinic Guidelines for Antimicrobial Use 2012–2013	Cleveland Clinic	USA	Americas	Physicians	The Cleveland Clinic Guidelines for Antimicrobial Use consists of tables describing laboratory tests, treatment guidelines for infections, and preferred antimicrobial use in various situations such as pregnancy and transplants. Topics addressed include cost-effectiveness, resistance patterns, antimicrobial usage data. *http://www.clevelandclinicmeded.com/medicalpubs/antimicrobial-guidelines/*	^a^
13	Décision+ / Décision+2	Université de LavalGovernment of Quebec	Canada	Americas	Physicians, residents	Decision+ (and Décision+2) integrates multiple educational/behavioral change components to promote shared decision making on antibiotic treatment options. Includes a Diagnostic Decision Support Tool for physician and patient to illustrate risks and benefits. It has been pilot tested, and embedded as a mandatory component of family medicine residency in Quebec. *http://www.decision.chaire.fmed.ulaval.ca/en/research/projects/decision/*	
14	Do Bugs Need Drugs?	Government of Alberta,Government of British Colombia	Canada	Americas	Healthcare professionals	Do Bugs Need Drugs is a community education project that includes resources and accredited courses for physicians and pharmacists, non-accredited courses for students and other professionals, and campaigns for the general public. Health professional students are involved in delivering the program to school children. *http://www.dobugsneeddrugs.org/about/#bc*	^a,b^
15	Get Smart	Centers for Disease Control and Prevention	USA	Americas	Healthcare professionals	The Get Smart for Healthcare program is a comprehensive program produced by the US CDC to improve antibiotic use. Resources for healthcare professional include a collection of program examples, Implementation resource, fact sheets, research evidence, video commentaries, and PowerPoint slides. General resources include video and radio public service announcements, pamphlets, posters, online resources, and more. This program includes Get Smart About Antibiotics Week, an annual awareness campaign complete with educational and promotional materials such as posters, graphics, press materials, and interactive activity ideas. *http://www.cdc.gov/getsmart/*	^a^
16	Get Smart About Antibiotics	Wake Forest School of Medicine;Centers for Disease Control and Prevention	USA	Americas	Medical students	Wake Forest University has developed a medical school curriculum around antimicrobial resistance. Several resources have been developed to assist with student learning including a series of video lectures and lecture notes, case studies and exam style questions. *http://www.wakehealth.edu/School/CAUSE/Get-Smart-About-Antibiotics.htm*	^a^
17	Get smart Colorado: use antibiotics wisely	Coalition	USA	Americas	Healthcare professionals, patients and the public	This campaign used pamphlets and posters, billboards, radio, television, website, letters, guidelines, and seminars to promote proper antibiotic use. Resources specifically for healthcare providers included newsletters, externally sourced treatment guides, pediatric academic detailing sheets, and links to external resources. Multiple resources were directed to healthcare providers but for use with patients, such as examination room posters and educational “prescription pads” for viral infections; these types of resources were availablein English and Spanish. The website also shares tips for non-prescription care and appropriate antibiotic use. *http://www.getsmartcolorado.com/*	^a^
18	Healthy Canadians Collection: Antibiotic Resistance	The Government of Canada	Canada	Americas	Healthcare professionals, patients and the public	The Government of Canada produced an online resource for bacteria, antibiotics, AMR, surveillance, and Canada’s efforts on these fronts. Resources for healthcare professionals include guidelines on infection management from The Public Health Agency of Canada, posters about reducing the spread of resistance in healthcare settings, and external resources such as Bugs and Drugs (see program #11). Other resources include educational videos, letters, infographics, posters, and pamphlets for the general public and for First Nations and Inuit peoples specifically. *http://www.healthycanadians.gc.ca/drugs-products-medicaments-produits/buying-using-achat-utilisation/antibiotic-resistance-antibiotique/index-eng.php*	^a^
19	Infectious Diseases Management Program at UCSF	University of California San Francisco	USA	Americas	Healthcare professionals	An inter-professional inter-hospital collaboration to better antimicrobial use and patient care. The program includes guidelines for antimicrobial therapy for various conditions, hospital-specific guidelines, and antimicrobial susceptibility information relevant to hospitals in San Francisco. *http://idmp.ucsf.edu/*	
20	Johns Hopkins Antimicrobial Stewardship Program	Johns Hopkins	USA	Americas	Physicians	Resource site for physicians including guidelines for acceptable and unacceptable use of antibiotics, antibiotic dosage and toxicity for adults, antifungals and vaccines, institution-specific guidelines and regulations, microbiology information, and treatment recommendations for specific infections. *http://www.hopkinsmedicine.org/microbiology/specimen/index.html http://www.hopkinsmedicine.org/amp/guidelines/Antibiotic_guidelines.pdf*	
21	Making a Difference in Infectious Disease Pharmacotherapy (MAD-ID) Canada	MAD-ID and University of Toronto	Canada	Americas	Pharmacists	A workshop held in Toronto in 2010, using an adapted version of the MAD-ID program that included additional content for the Canadian context. Topics included de-escalation and streamlining, and Making Antimicrobial Stewardship work. *Courtesy of Linda Dresser at University Health Network in Toronto*	
22	Making a Difference in Infectious Disease Pharmacotherapy (MAD-ID) USA	MAD-ID	USA	Americas	Pharmacists	MAD-ID offers a basic and an advanced antimicrobial stewardship training program which are designed for pharmacists and physicians. Continuing education credit is available for pharmacists in the USA. *http://mad-id.org/antimicrobial-stewardship-programs/*	^b^
23	Michigan antibiotic resistance reduction coalition	Coalition	USA	Americas	Healthcare professionals, patients and the public	This state campaign provided information about antimicrobials and resistance to healthcare providers and the public using TV and radio announcements, pamphlets, educational cartoons for children, and posters. Healthcare professionals were targeted specifically with infographics, and pamphlets about prescribing and communicating with patients. A webinar on antibiotics in upper respiratory tract infections was also promoted to healthcare providers. *http://www.mi-marr.org/*	^a^
24	Multipronged Education Strategy on Antibiotic Prescribing in Quebec	INESSS Quebec	Canada	Americas	Physicians, pharmacists, medical residents	Educational mailout with guidelines targeting the most common conditions in outpatient settings, clinical information on diagnosis and antibiotic recommendations. The emphasis was on proper antibiotic regimens and not using antibiotics when viral infections were suspected, plus using shorted possible treatment. Guidelines were produced by the Quebec Medication Council in collaboration with physicians and pharmacists. A letter of support from all key stakeholders (MOH, College of Physicians, College of Pharmacists, Medical Associations) accompanied the mail-out. *Weiss* et al. *Clin Infect Dis 2011; 53(5):433–9. https://www.ncbi.nlm.nih.gov/pubmed/21791439*	
25	National information program on antibiotics	Coalition	Canada	Americas	Physicians, pharmacists, patients and the public	This program uses pamphlets and posters, print media, radio and letters to summarize information on antibiotics, bacterial infections, antibiotic use, and resistance. *http://www.antibiotics-info.org*	^a^
26	NCCID Resources for Healthcare Providers Website (Antibiotic Awareness Week Webinar Archives and NCCID Resources for healthcare providers)	National Collaborating Centre for Infectious Diseases	Canada	Americas	Healthcare professionals	The National Collaborating Centre for Infectious Diseases (NCCID) website provides free online resources focused on antibiotics and AMR. The Antibiotic Awareness Week Webinar Archives is an online resource that compiles YouTube videos, transcripts, and presentation slides from previous seminars. Resources are available in English or French and online access is free. Resources for Healthcare Providers include factsheets, prescription sheets, a script outlining how to communicate with patients, treatment guidelines. *http://nccid.ca/antibiotic-awareness/ http://nccid.ca/antibiotics-and-health-care-providers/#patients*	
27	Nebraska appropriate antibiotic use campaign	Regional department of health	USA	Americas	Healthcare professionals, patients and the public	A state antibiotic awareness campaign. Resources for healthcare providers include links to local AMR stakeholder organizations such as the Nebraska Infection Control Network, and other external resources such as links to CDC resources. *http://dhhs.ne.gov/publichealth/HAI/Pages/Resistance.aspx*	^a^
28	Nursing Home Antimicrobial Stewardship Modules	Agency for Healthcare Research and Quality, Department of Health and Human Services	USA	Americas	Nurses	Nursing Home Antimicrobial Stewardship Guide provided by the Agency for Healthcare Research and Quality seeks to train nurses and other nursing home staff on AMR, treatment decisions. The program provides toolkits on antimicrobial stewardship and antibiotic treatment which include informational handouts, online training resources, educational PowerPoint slides, and links to academic articles. *http://www.ahrq.gov/professionals/quality-patient-safety/patient-safety-resources/resources/nh-aspguide/index.html*	
29	Oregon alliance working for antibiotic resistance education	Coalition	USA	Americas	Healthcare professionals, patients and the public	This state coalition seeks to promote careful use of antibiotics by targeting healthcare providers, and the general public. Provider resources include posters, continuing education course links, prescribing statistics, treatment guidelines, and seminars and the CDC’s Get Smart week are promoted. Resources for the public include activity books for children, brochures and information sheets about prudent antibiotic use and resistance. *https://public.health.oregon.gov/DiseasesConditions/CommunicableDisease/AntibioticResistance/Pages/index.aspx*	^a^
30	Public Health Ontario Antimicrobial Stewardship Program	Public Health Ontario	Canada	Americas	Doctors, nurses, pharmacists	Public Health Ontario provides resources such as posters to promote appropriate prescribing in hospital settings, presentation slides on AMR and stewardship, webinars, and print guidelines. These resources are shared to support acute care facilities in meeting Accreditation Canada’s mandate requiring AMS programs. *https://www.publichealthontario.ca/en/BrowseByTopic/InfectiousDiseases/AntimicrobialStewardshipProgram/Pages/Antimicrobial-Stewardship-Program.aspx*	^a^
31	South Carolina Careful Antibiotic Use (CAUse)	South Carolina Department of Health and Environmental Control	USA	Americas	Healthcare professionals, patients and the public	An adapted version of the CDC Get Smart program which initially targeted HCW for education on diagnostic and treatment guidelines, then targeted parents of young children about appropriate use. *Overview is available in this report: http://www.scstatehouse.gov/Archives/DHEC/AROStrategicPlan-final1-15-07dr_3.pdf*	^a^
32	Stop antibiotic misuse in Minnesota	Coalition	USA	Americas	Healthcare professionals, patients and the public	This state program used pamphlets and posters, letters, guidelines, seminars and a childcare curriculum. The website provides links for professionals include policy statements, statistics, journal articles and guidelines relating to AMR and infections. *http://www.minnesotaarc.org/*	^a^
33	Stop the resistance—use antibiotics wisely	Nevada Public Health Foundation	USA	Americas	Healthcare professionals, patients and the public	This state educational campaign seeks to reach healthcare providers the public to encourage and appropriate antibiotic use. This campaign provides resources including clinical practice guidelines, resources from the CDC, and information sheets informing prescribers about appropriate antibiotic use. General resources include educational brochures on AMR, colds, and infections in English and Spanish, and links to websites and games for children’s education on topics such as handwashing and bacteria. *http://www.nevadapublichealthfoundation.org/programs/nevadans-for-antibiotic-awareness/*	^a^
34	The California Antimicrobial Stewardship Program Initiative	California Department of Public Health	USA	Americas	Healthcare professionals	This program shares resources associated with the California Department of Public Health, such as an antimicrobial stewardship webinar series directed to nursing homes, and a compilation of example stewardship programs. The focus of this program is to support the implementation of stewardship programs in California hospitals and healthcare facilities. *http://www.cdph.ca.gov/programs/hai/Pages/AntimicrobialStewardshipProgramInitiative.aspx*	
35	University of Pennsylvania Antimicrobial Stewardship	University of Pennsylvania	USA	Americas	Medical students, residents	This educational webpage includes presentation slides, PDF handouts, and lecture recordings relevant to antimicrobial education for medical students and residents. Topics include drug availability, prophylaxis, as well as University-specific stewardship policies, antibiotic prescribing recommendations, restrictions and economic considerations. *http://www.uphs.upenn.edu/antibiotics/*	^a^
36	Washington state’s campaign to fight antibiotic resistance	Regional department of health	USA	Americas	Physicians, patients and the public	This state health department website compiled links to publications and external resources on AMR for patients, physicians, and educators. Resources for the public include school-based awareness programs, information sheets, and links to the FDA and CDC. *http://www.doh.wa.gov/YouandYourFamily/IllnessandDisease/AntibioticResistance*	^a^
37	Awareness week, lectures and panel discussions	Students for Antimicrobial Stewardship Society	Canada	Americas	Healthcare students	This student society has chapters in medical, dental and pharmacy schools across Canada. They have organized an annual awareness week with lectures and panel discussions to educate students about AMR and AMS. *http://www.sass-canada.ca/*	
38	Antibioclic: Antibiotherapie rationelle en soins primaires	Societe de Pathologie Infectieuse de Langue Francaise/French Infectious Diseases Society (SPILF)	France	Europe	Physicians	This website offers therapeutic recommendations to primary care physicians. Physicians input their patients’ pathology and other clinical considerations, and the website provides recommended antibiotic treatment with considerations and options for allergies and contraindications, as well as brief information about the antibiotics. The website also shares news related to infections and antibiotics. *http://antibioclic.com/*	^a^
39	Antibioguide and Antibioville	Antibiolor	France	Europe	Physicians	The Antibiolor guidelines were published to assist physicians in the management of infections: Antibioguide (2005) for hospital physicians and Antibioville (2004) for community physicians. The guidelines are available online, in print, and through an app. Antibiolor also provides Antibiotel, a telephone advice service for physicians requiring advice regarding management of patients with infectious diseases. *http://www.antibiolor.org/*	^a^
40	Antibiotic advice in Île-de-France	Societe de Pathologie Infectieuse de Langue Francaise/French Infectious Diseases Society (SPILF)	France	Europe	Physicians	Telephone advice relating to antibiotics was provided by physicians for physicians; *Wang* et al. *in Med Mal Infect. 2015; 45(4):111–23.* *https://www.ncbi.nlm.nih.gov/pubmed/25747501*	
41	Antibiotic Awareness Campaign	European Phamacy Students Association	Multiple	Europe	Pharmacy students	The European Pharmacy Students Association created an Antibiotic Awareness Campaign in order to raise awareness about antibiotic use, decrease demand for antibiotics, and to educate the public on AMR. The EPSA created a campaign for their assembly in 2013 and a digital campaign was developed in partnership with the ECDC for the European Antibiotic Awareness Day. *http://www.epsa-online.org/index.php/projects/public-health-campaigns/previous-campaigns*	^a^
42	Antibiotic Guardian	Public Health England	United Kingdom	Europe	Healthcare professionals, patients and the public	Antibiotic Guardian consists of a collection of pledges with over 30,000 signatures directed to healthcare professionals, students, and the general public. Resources for healthcare professionals include links to country-specific EAAD and WAAW resources and public resources include patient stories, posters, leaflets, and links to other programs including WAAD, e-bug, and other informational websites. *http://antibioticguardian.com/*	^a^
43	Antibiotic Resistance in Primary Care E-Module	Royal College of General Practitioners Learning	United Kingdom	Europe	Healthcare professionals	This hour long continuing professional education course for primary care physicians in the UK looking to learn about antibiotic prescribing, resistance, and resources for practice. *http://elearning.rcgp.org.uk/course/info.php?popup=0&id=167*	^b^
44	Antibiotic Resistance: The Silent Tsunami	Uppsala University	Sweden	Europe	Healthcare professionals	This 4-week-long online course discusses the spread of antibiotic resistance, the development of antibiotics, the current state of resistance and antibiotic use, and actions to tackle AMR. This course is free and composed of 3 h per week of online content. *https://www.futurelearn.com/courses/antibiotic-resistance*	^a^,^b^
45	Antibiotics only when necessary (Le Projet Gepie: Groupe d’Etude et de Prévention des Infections de l’Enfant)	Coalition: academic centers, health insurance, medical association, institute for public health	France	Europe	Physicians	This campaign employed pamphlets and posters, website, letters, guidelines, and academic detailing. The program’s website contains resources such as guidelines for antibiotic use or disuse, progression of conditions such as bronchitis, in addition to general guidelines and guidelines for antibiotic use in children. A list of publications by the GEPIR group is provided. This group also supports an initiative for teacher and youth education about microorganisms, infections, and their treatment known as “Projet E-bug”. *http://www.gepie.org/*	^a^
46	Antibiotics, use them in an adequate way	Coalition: pharmaceutical industry, department of health, professional organizations	Portugal	Europe	Healthcare professionals, patients and the public	A seasonal campaign to reduce antibiotic use in Portugal that used several intervention types to target a wide range of professionals and the public. *Huttner* et al. *in Lancet Infect Dis. 2010; 10(1):17–31. https://www.ncbi.nlm.nih.gov/pubmed/20129146*	
47	Antimicrobial Resistance and Healthcare Associated Infections Foundation Course on Hospital Infection Control	Healthcare Infection Society	United Kingdom	Europe	Residents	This 3 day long in-person course provides information on healthcare associated infections and AMR, developing surveillance programs, outbreak investigation, hospital hygiene, the basics of antimicrobial stewardship, and healthcare associated infections. It can be taken as a stand-alone course or as part of a Masters or Diploma course. *https://www.his.org.uk/education/courses/*	^b^
48	Antimicrobial Resource Centre	British Society for Antimicrobial Chemotherapy	United Kingdom	Europe	Healthcare professionals	This database of resources compiles many educational materials and links including links to open online courses, audio, guidelines, images, press articles, publications, posters, research papers, slide sets, systematic reviews and video lectures on diverse topics relating to antimicrobials and resistance. *http://stage.bsac-arc.com/#*	^a^
49	Antimicrobial Stewardship Educational Workbook	Scottish Antimicrobial Prescribing Group	United Kingdom	Europe	Nurses, midwives	This interactive workbook contains question prompts and links to informational videos to support learning around antimicrobial stewardship, specifically including chapters on stewardship in practice, bacteria, resistance and antibiotics, assessment of infection, and hospital practice. *http://www.scottishmedicines.org.uk/files/sapg/Final_Antimicrobial_Stewardship_Print_Wkb_Jan_2016.pdf*	^a^
50	Antimicrobial stewardship: systems and processes for effective antimicrobial medicine use	National Institute for Health and Care Excellence	United Kingdom	Europe	Healthcare professionals, patients and the public	The National Institute for Health and Care Excellence publishes guidelines including Antimicrobial Stewardship. These guidelines include recommendations for the implementation of stewardship programs, antimicrobial prescribing, and new antimicrobials. *https://www.nice.org.uk/guidance/ng15*	
51	Antimicrobial Susceptibility Testing and Surveillance: from Laboratory to Clinic.	European Society of Clinical Microbiology and Infectious Diseases	Germany	Europe	Clinical microbiologists	This 3-day-long workshop consists of a series of lectures and laboratory sessions that cover European Committee on Antimicrobial Susceptibility Testing (EUCAST) clinical breakpoints, antimicrobial susceptibility testing, antimicrobial surveillance systems, and antimicrobial guidelines. This is part of a series of microbiology workshops organized by the ESCMID. *https://www.escmid.org/profession_career/educational_activities/escmid_courses_and_workshops/antimicrobial_susceptibility_testing_and_surveillance_a_eucast_esgars_and_epasg_perspective/*	
52	Antimicrobials and infection management	Centre for Pharmacy Postgraduate Education	United Kingdom	Europe	Pharmacists, pharmacy students,	This 10-h e-learning course focuses on describing the action of antimicrobials, selecting appropriate antimicrobial agents, antimicrobial prophylaxis, patient treatment, and healthcare-associated infections. This program is only available to members of the General Pharmaceutical Council. *https://www.cppe.ac.uk/programmes/l/antimicro-e-01/*	
53	Bugs and antibiotics: the role of the Greek Physician in the fight against microbial resistance	University of Athens	Greece	Europe	Physicians	A regional education campaign in 2009/10 organized by the medical school at the University of Athens. A series of meetings were organized with primary care physicians followed by 4 interactive case study sessions. Participants also received a set of guidelines and a booklet on management of community acquired infections. *Plachouras* et al. *in BMC Public Health 2014;14:866.* *https://www.ncbi.nlm.nih.gov/pmc/articles/PMC4148920/*	
54	CNAMTS Program	National Health Insurance Fund for Salaried Workers (CNAMTS)	France	Europe	Physicians	The National Health Insurance Fund for Salaried Workers’ (CNAMTS) work for AMR includes holding meetings with Delegates of the National Health Insurance (DAM) and physicians. Information sheets and recommendations on medical practice are handed out to physicians during these meetings, which are held 3 or 4 times a year. *Huttner* et al. *in Lancet Infect Dis. 2010; 10(1):17–31.* *https://www.ncbi.nlm.nih.gov/pubmed/20129146*	
55	Collection: Antimicrobial Resistance (AMR)	National government	UK	Europe	Healthcare professionals,	The UK government supports an online collection of resources relating to AMR and inks to research publications, technical and clinical guidelines, posters, research, news updates, and strategic documents such as the UK AMR strategies and progress reports. One noteworthy resource is the “Antimicrobial Resistance Resource Handbook” which provides information on policy, recommendations, education, tools, surveillance, and other resources that support stewardship and AMR reduction. *https://www.gov.uk/government/collections/antimicrobial-resistance-amr-information-and-resources*	^a^
56	Correct use antibiotics	Belgian Antibiotic Policy Coordination Committee (BAPCOC)	Belgium	Europe	Healthcare professionals, patients and the public	This program’s webpages offer links to academic articles are directed towards healthcare professionals. Webpages answer basic questions (e.g. what are antibiotics, asking advice from your healthcare provider), promote children’s educational comics on antimicrobials and infections, and provide links to external resources for the general public. The program also includes radio commercials. Many resources are available in French, English, Dutch and German. *http://www.correctuseantibiotics.be/en*	^a^
57	ESCMID Summer School	European Society of Clinical Microbiology and Infectious Diseases	Spain	Europe	Physicians, clinical microbiologists	This week long in-person course focuses on clinical microbiology and infections. Topics include infection management, therapy beyond antibiotics, viral infections, multidrug resistance, international health and career development. *https://www.escmid.org/dates_events/escmid_summer_school/*	
58	ESCMID/ASM Conference on Drug Development to Meet the Challenge of Antimicrobial Resistance	European Society of Clinical Microbiology and Infectious Diseases	Austria	Europe	Healthcare professionals	This 3-day conference consists of a series of lectures, symposiums, debates, round table discussions, and interactive question and answer sessions relating to AMR. Topics include clinical use of off-patent antibiotics, future areas of research, breakpoints, and changing antimicrobial administration and guidelines. *https://www.escmid.org/research_projects/escmid_conferences/escmidasm_conference/*	
59	European Antibiotic Awareness Day	European Centre for Disease Prevention and Control		Europe	Healthcare professionals, patients and the public	European Antibiotic Awareness Day promotes education about antibiotic use and resistance annually on November 18th. The website contains diverse downloadable resources for prescribers as well as patients and the public. Toolkits for prescribers include presentations for in-hospital training on AMR, patient dialog guides and factsheets. *http://ecdc.europa.eu/en/eaad/Pages/Home.aspx*	^a^
60	General Practitioner conferences to reduce over-prescribing	Health insurance office, Créteil hospital center	France	Europe	Physicians	Conferences and meetings involved a discussion of prescribing profiles and promoted awareness of antibiotic use. *Wang* et al. *in Med Mal Infect. 2015; 45(4):111–23.* *https://www.ncbi.nlm.nih.gov/pubmed/25747501*	
61	Interdisciplinary Regional Program for the Control of antimicrobial resistance (PRIMAIR)		France	Europe	Physicians	This program promoted continuing education and antibiotic treatment guidelines for general practitioners in the Franche-Comté region of France. *Wang* et al. *in Med Mal Infect. 2015; 45(4):111–23.* *https://www.ncbi.nlm.nih.gov/pubmed/25747501*	^b^
62	MedQual	Centre Ressource en Antibiologie	France	Europe	Healthcare professionals, patients and the public	This network is dedicated to monitoring antibiotic use and antibiotic resistance. The website provides online advice and decision trees for physicians managing infectious diseases. It also publishes a monthly electronic newsletter. Telephone advice to healthcare professionals is provided. *http://www.medqual.fr/*	^a^
63	National Health Service Scotland Education and Training	National Health Service Scotland/NHS Education for Scotland	United Kingdom	Europe	Physicians, nurses, pharmacists	The Scottish Reduction in Antimicrobial Prescribing (ScRAP) Programme is an educational toolkit under this program to encourage a reduction in unnecessary antibiotic prescribing. ScRAP includes teaching topics and lesson plans for local facilitators, video lectures, and videos of patient-scenarios and other teaching materials. Some programs include clinical vignettes and multiple choice assessments. Other resources include interactive antimicrobial stewardship educational textbooks/workbooks targeted towards nurses and midwives, complete with information, best practice examples, evaluation questions, and reflection prompts, as well as PowerPoint presentation slides for practitioner education. *http://www.nes.scot.nhs.uk/education-and-training.aspx*	
64	Nice France teaching hospital telephone advice service	Nice teaching hospital Infectious Disease Department	France	Europe	Physicians	This service consisted of telephone advice with the option of in person consultations for general practitioners in the Provence-Alpes-Côte d’Azur Est region of France. Was terminated due to lack of resources. *Wang* et al. *in Med Mal Infect. 2015; 45(4):111–23.* *https://www.ncbi.nlm.nih.gov/pubmed/25747501*	
65	PrescQIPP Antimicrobial Stewardship Webkit	PrescQIPP	United Kingdom	Europe	Healthcare professionals	This webpage provides resources and links to educate healthcare providers about antimicrobial stewardship and AMR. Types of resources include data on infections, infographics, guidelines relating to AMR, information about diagnoses, and online links. *https://www.prescqipp.info/antimicrobial-stewardship/projects/antimicrobial-stewardship#diagnostics*	^a^
66	Project children and antibiotics—for appropriate antibiotic use in children	Regional department of health	Italy	Europe	Physicians	This program targeted antibiotic prescribing to pediatricians by running seminars and adapting “Do Bugs Need Drugs” (see program #14). *Huttner* et al. *in Lancet Infect Dis. 2010; 10(1):17–31.* *https://www.ncbi.nlm.nih.gov/pubmed/20129146*	
67	Reducing Antimicrobial Resistance	E-Learning for Healthcare, Health Education England	United Kingdom	Europe	Healthcare professionals, patients and the public	This program consists of an e-learning session, short videos, and links to other resources such as Antibiotic Guardian (see program #42) and Future Learn MOOC (see program #90). The objective is to discuss risks and development of AMR, the misuse of antibiotics, and improve responses to patients. *http://www.e-lfh.org.uk/programmes/antimicrobial-resistance*	^a^
68	STAR: Stemming the Tide of Antibiotic Resistance	Healthcare CPD	United Kingdom	Europe	Physicians	This hour long training intervention informs medical practitioners about antibiotic prescription, resistance, and to encourage behavioral change and reflection about current practices. *http://www.healthcarecpd.com/course/star-stemming-the-tide-of-antibiotic-resistance*	^a^
69	Strama	The Swedish Strategic Programme Against Antibiotic Resistance (Strama)	Sweden	Europe	Healthcare professionals	The Swedish Strategic Programme Against Antibiotic Resistance (Strama) shares action plans, scientific research protocols and data. They link to other resources, such as online courses for doctors and nurses. The National Strama Days are annual awareness campaigns meant to provide education on antibiotic issues including resistance and policymaking and facilitate networking. *http://strama.se/?lang=en See report: “Swedish work on containment of antibiotic resistance” http://strama.se/examples-of-projects/?lang=en*	
70	Strategic and Exploratory Workshop	Joint Programming Initiative on Antimicrobial Resistance (JPIAMR)	Norway	Europe	Healthcare professionals	Joint Programming Initiative on Antimicrobial Resistance (JPIAMR) facilitates a series of workshops. These workshops revolve around selected keynote lectures, breakout sessions and plenary discussions. Workshop titles include “The Interplay Between AMR Surveillance and Science” and “Early Discovery of New Antibiotics”. *http://www.jpiamr.eu/activities/the-interplay-between-amr-surveillance-and-science/*	
71	SWAB (Dutch Working Party on Antibiotic Policy)	Society of Infectious Diseases	The Netherlands	Europe	Healthcare professionals	The Dutch Working Party on Antibiotic Policy (SWAB) has developed numerous guidelines relating to antibiotics and AMR focusing on general information, specific infections, MRSA, and antibiotic prophylaxis. The group also holds an annual symposium on AMR and contributes to EAAD online resources. *http://www.swab.nl/swab/cms3.nsf/viewdoc/102D6425C642468DC125757F0039CDDE*	
72	The Regional Functional Unit of Infectious Diseases (UFIR)	Ajaccio hospital directorate, Corsica regional health authority	France	Europe	Physicians	This unit offered telephone advice for general practitioners in Corsica as well as post-graduate education on antibiotics. *Wang* et al. *in Med Mal Infect. 2015; 45(4):111–23.* *https://www.ncbi.nlm.nih.gov/pubmed/25747501*	
73	Treat Antibiotics Responsibly, Guidance, Education, Tools (TARGET) Antibiotics Toolkit	Royal College of General Practitioners, Public Health England	United Kingdom	Europe	Healthcare professionals	This toolkit includes antibiotic prescribing data, toolkits for conducting audits, posters and pamphlets for patients, guidelines from Public Health England, resources to support implementation of the TARGET toolkit, and links to external resources and stakeholders relevant to AMR and antibiotics. Resources for general practitioners specifically include a self-assessment checklist evaluating good antimicrobial practices. Training resources include a webinar series, presentation templates and notes, and links to two e-modules. The webinar series runs from November to December 2016 and provides information on how to improve prescribing and patient care. *http://www.rcgp.org.uk/TARGETantibiotics*	^a^
74	URPS Program	Regional health authority	France	Europe	Physicians	As a local initiative, information sheets were distributed to general practitioners at clinical presentations. Information sheets related to infection management, clinical presentation, and antibiotic prescribing for patients aged 15 to 65. *Wang* et al. *in Med Mal Infect. 2015; 45(4):111–23.* *https://www.ncbi.nlm.nih.gov/pubmed/25747501*	
75	Antimicrobial Stewardship: Principles and Practice	European Society of Clinical Microbiology and Infectious Diseases		Europe	Healthcare professionals	This course will discuss basic principles of AMR and consumption surveillance, implementation of AMS programs, how to change prescriber behaviors, and how to integrate AMS education in student, resident and postgraduate education at an institutional level. Planned for 2017. *https://escmid.pulselinks.com/event/13464*	
76	Antibiotic campaign	Health maintenance organization	Israel	Middle East	Physicians	A seasonal campaign to distribute guidelines and information on resistance to physicians, with a mass media component. *Huttner* et al. *in Lancet Infect Dis. 2010; 10(1):17–31.* *https://www.ncbi.nlm.nih.gov/pubmed/20129146*	^a^
77	Antibiotics Smart Use: Thailand	Antimicrobial Resistance Containment Program (AMRCP) in Thailand	Thailand	South East Asia	Healthcare professionals, patients and the public	Country-wide campaign that educated prescribers about antibiotics in half-day long in-hospital training sessions. Training included sharing experiences, learning about treatment guidelines and diagnostic tools, as well as instructions on implementing resources for patient education such as DVDs, pamphlets and other materials. *http://www.reactgroup.org/toolbox/thailands-work-to-limit-abr/#zp-ID-2628-280251-WJR8EDEQ*	
78	ASPIC (Antibiotic Stewardship, Prevention of Infection & Control)	Indian Council of Medical Research	India	South East Asia	Clinical pharmacists, clinical microbiologists	This workshop taught skills and knowledge about infection prevention and control, implementation of antimicrobial policy guidelines for rational use of antibiotics to curb resistance, conducting research projects in antibiotic policy. Workshop consisted of 5 days of lectures, site visits, practical training, demonstrations and project discussions. *Chandy* et al. *in Indian J Med Res 2014; 139(2):226–230. https://www.ncbi.nlm.nih.gov/pmc/articles/PMC4001333/*	
79	AMR Training	Songdo Hospital	Mongolia	Western Pacific	Physicians	Quarterly Education sessions and surveys of antimicrobial prescribing in hospital. *Courtesy of WHO WPRO Office: WHO Mongolia Country Report May 2016 by Koning and Peel*	
80	Antibiotic Awareness Week	Government of Viet NamWHO	Viet Nam	Western Pacific	Healthcare professionals, patients and the public	This awareness week campaign included a pledge initiative, public outreach events, posters and banners in hospitals, and social media use. *http://www.wpro.who.int/vietnam/mediacentre/releases/2015/antibiotics_resistance_week2015/en/* *https://www.facebook.com/amrweekvietnam/*	^a^
81	DOH Manual of Procedures for Implementing AMS in Hospitals	Department of Health (Philippines)World Health Organization	Philippines	Western Pacific	Physicians	The DOH Manual of Procedures for Implementing AMS in Hospitals is an operational guide that is also used as a reference for developing training programs and materials. *Information courtesy of the WHO WPRO regional office.*	^a^
82	National Prescribing Service: Medicinewise Resource Website	National Prescribing Serivce (NPS): Medicinewise	Australia	Western Pacific	Healthcare professionals	The NPS website includes resources and online learning modules. The website of antibiotic resources for health care providers includes tools for consultation with patients, pamphlets and posters, guidelines. Five online case study-based modules are available for general practitioners, nurses, and healthcare professional students on the topics of antimicrobial prescribing, bacterial infections, urinary tract infections, pneumonia, and surgical antibiotic prophylaxis. These modules are recognized by The Royal Australian College of General Practitioners as fulfilling some requirements of their Continuing Professional Development program as well as by the Australian College of Rural and Remote Medicine for some of their Professional Development Program requirements. *http://www.nps.org.au/medicines/infections-and-infestations/antibiotics* *http://www.nps.org.au/health-professionals/cpd*	^a,b^
83	Training-of-Trainers Workshop on the Antimicrobial Stewardship Advocacy Training	Department of Health (Philippines)World Health Organization (WPRO)	Philippines	Western Pacific	Healthcare professionals	The Training-of-Trainers was delivered by a representative from NPS Medicinewise (Australia). This consisted of an interactive session on current initiatives and challenges of each hospital in relation to infection prevention and control, rational use of antimicrobials and surveillance of AMR. Also included was a consultation on draft AMS pilot program implementation. *http://www.ncpam.doh.gov.ph/index.php/en/training-of-trainers-workshop-on-the-antimicrobial-stewardship-advocacy-package*	
84	Training-workshop for government Level III hospitals on the implementation of the AMS	Department of Health (Philippines)World Health OrganizationCorazon Locsin Montelibano Memorial Regional Hospital	Philippines	Western Pacific	Physicians	This workshop began as a pilot program at Philippine General Hospital and Corazon Locsin Montelibano Memorial Regional Hospital. An AMS program with an educational component was tested and has now been scaled up to provide training at all DOH Level III hospitals. *Information courtesy of the WHO WPRO regional office.*	
85	WHO WPR Pledge to use antibiotics responsibly	World Health Organization Western Pacific Region		Western Pacific	Healthcare professionals	This pledge designed by the World Health Organization Western Pacific regional office discusses a commitment to the use of antibiotics as prescribed, good hygiene, and encouraging responsible use in friends and family. Includes information on AMR. *https://apps.wpro.who.int/amr/pledge.aspx*	^a^
86	Win the War Against AMR	Department of Health	Philippines	Western Pacific	Healthcare professionals	Win the War Against AMR is a pledge for healthcare providers to use antimicrobials responsibly. Pledge documents comment upon judicious use of antimicrobials, continuing education of others, promoting preventative measures, and supporting research of antimicrobial drugs and diagnostic technologies. *http://www.ncpam.doh.gov.ph/index.php/en/major-program?id=52*	^a^
87	Medication Shortage Fact Sheets and Infographics	National Centre for Antimicrobial Stewardship (NCAS)	Australia	Western Pacific	Healthcare professionals	This resource provides fact sheets and infographics designed in response to recent problems with the supply of antimicrobials in Australia. The fact sheets provide advice and possibilities for alternative therapy when vital medications are in short supply. The infographics provide information on antimicrobial prescribing in various healthcare facilities. Both resources are easily accessible online. *https://www.ncas-australia.org/education*	^a^
88	AMS Implementation Toolkit	Clinical Excellence Commission	Australia	Western Pacific	Healthcare professionals	The Clinical Excellence Commission has developed resources to support the implementation of hospital AMS programs that embody the core elements required for program implementation and maintenance. These resources also facilitate to identify gaps present in currently established AMS programs. *http://www.cec.health.nsw.gov.au/patient-safety-programs/medication-safety/antimicrobial-stewardship/quah/ams-implementation-toolkit*	
89	Antibiotic Stewardship Clinical Care Standards	Australian Commission on Safetey and Quality in Health Care	Australia	Western Pacific	Healthcare professionals	This resource provides guidance to clinicians and health service managers on delivering appropriate care when prescribing antibiotics. The resource also provides a set of suggested indicators to assist with local implementation of the Antimicrobial Stewardship Clinical Care Standard. Clinicians and health services can use the indicators to monitor implementation of the quality statements. *https://www.safetyandquality.gov.au/our-work/clinical-care-standards/antimicrobial-stewardship-clinical-care-standard/*	^a^
90	Antimicrobial Stewardship: Managing Antibiotic Resistance	University of Dundee;British Society for Antimicrobial Chemotherapy	United Kingdom	Global	Healthcare professionals	This 6-week-long Massive Open Online course focuses on improving antimicrobial prescribing and AMS in hospital settings and can be applied to other settings. The course has run twice on the FutureLearn platform and involves readings, case study videos, video interviews with experts, and online discussion with peers and educators. Course topics include an overview of resistance as a global problem, the importance and key elements of antimicrobial stewardship, measurement of antibiotic prescribing, emerging strategies for antimicrobial stewardship, behavior change, and the importance of collaboration and coordination. *https://www.futurelearn.com/courses/antimicrobial-stewardship*	^a,b^
91	Basic Concepts Book	International Federation of Infection Control	United Kingdom	Global	Healthcare professionals	This book, Basic Concepts, by the International Federation of Infection Control contains a chapter on “Principles of Antibiotic Policies”. Key topics in this chapter include the rational use of antibiotics to combat resistance, the role of AMR stewardship programs, targeted antibiotic prescribing and treatment, and microbiology laboratory services in relationship to AMR. It is available in English, Arabic, French, Spanish, Italian, Bulgarian and Hungarian. *http://theific.org/basic-concepts-english-version-2016/*	^a^
92	Basic Infection Control Training Programme Course	International Federation of Infection Control	United Kingdom	Global	Healthcare professionals	This infection control course contains a module that focuses explaining how antibiotic use selects strains of bacteria, identifying mechanisms of AMR stewardship programs, the role of microbiology laboratories in resistance, as well as healthcare associated infections and outbreaks in general. *http://theific.org/basic-ic-training-course-outline/*	^a^
93	Coursera Course: Antimicrobial resistance - theory and methods	Technical University of Denmark	Denmark	Global	Healthcare professional	This 5 week long online MOOC teaches on antimicrobial action, antimicrobial resistance, antimicrobial susceptibility testing, interpretation, quality assurance, and bacterial genomic testing through online videos lectures and graded quizzes. *https://www.coursera.org/learn/antimicrobial-resistance*	^a^
94	ReAct Toolbox	Uppsala University	Sweden	Global	Healthcare professionals	Resource website on AMR and AMS with profiles of useful interventions. *http://www.reactgroup.org/toolbox/*	^a^

^a^The resource was available free of charge

^b^The resource was for continuing professional development credit or provided a certificate of completion or counted towards a degree program

We identified 24 multi-component umbrella programs, which typically included web-based resources, videos, references to guidelines, and promotional materials, such as posters, pamphlets, or infographics. Programs that incorporated four or more program types were defined as umbrella programs. Umbrella programs tended to have broader target audiences and wider scopes; in many cases, their awareness campaign materials were targeted towards a general audience, while online reading and guidelines were more often aimed at HCWs. Many umbrella programs targeted their messaging at HCWs—including physicians, pharmacists, and sometimes nurses—as a group, rather than targeting professions or specialties individually.

**Fig. 1:**
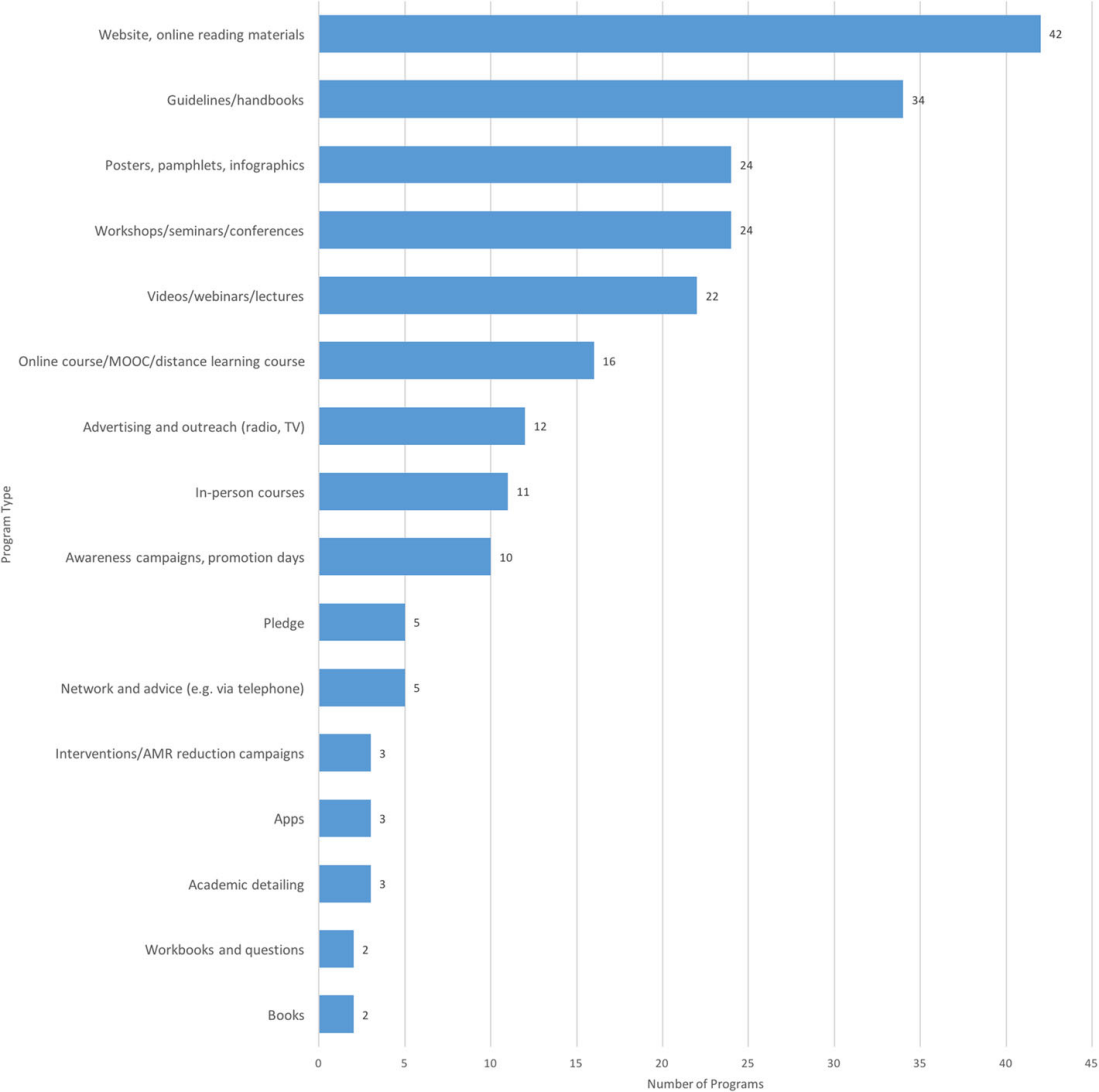
Number of identified AMR education programs by program type

Of the programs identified (see Fig. 1), the most common program type was websites; typically, however, online resources were only part of a larger program. This is unsurprising as online resources are effective platforms for sharing other program types, including videos, guidelines, pamphlets, and workbooks. Guidelines and handbooks were also a common resource, often shared online in government and hospital education initiatives; these described information about key patient populations and prescribing guidelines and were intended as reference resources. By contrast, we also identified a small number of workbooks and question sets intended as a teaching resource.

Other teaching resources available online included videos, webinars, and lecture slides. Online courses, including massive open online courses (MOOCs), are becoming common and often incorporate online videos and discussion boards to engage participants. In Fall 2016, there were two MOOCs on the FutureLearn platform (see Initiatives #44 and #90). The MOOC run by the University of Dundee and British Society for Antimicrobial Chemotherapy (Initiative #90) has gained global popularity. Several experts referenced this course as a resource used in their country; the course has been offered three times to date and participants have come from more than 41 countries.

**Fig. 2:**
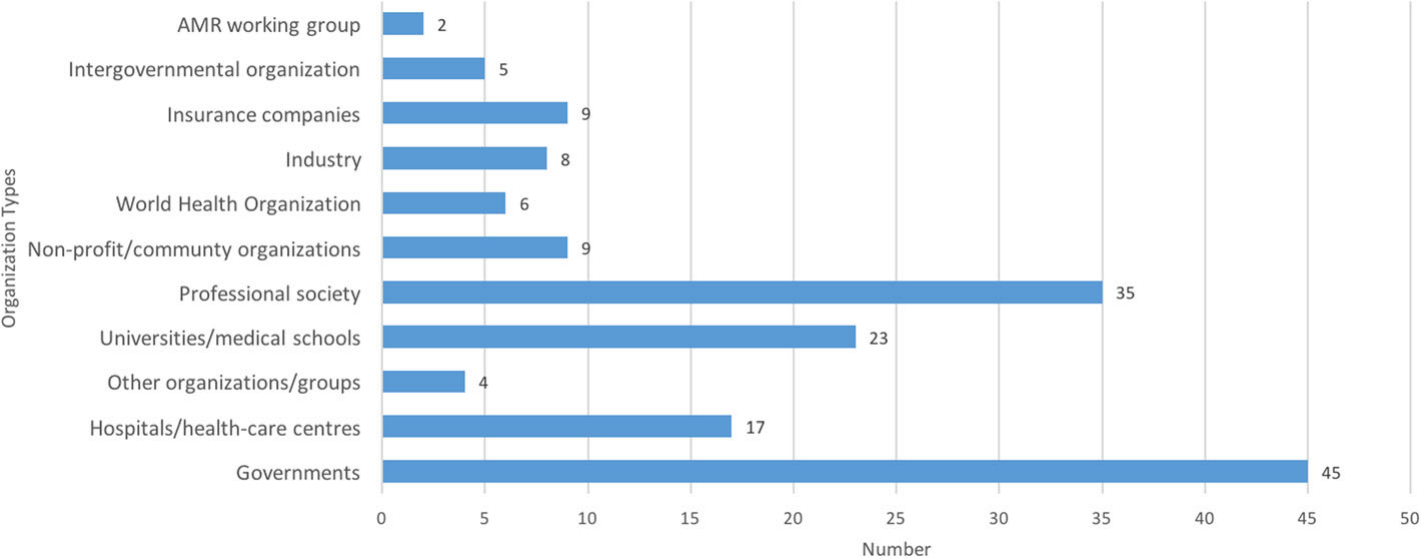
Number of identified AMR education programs by organization type

Figure 2 shows participating organizations grouped by type. Overall, the most commonly identified organizations were governmental bodies, often the national/provincial/state-level body responsible for health and/or education. We also found that health insurance companies often partnered with other organizations in the USA to support AMR education campaigns. Collaborations with industry came almost entirely from North America. Outside of the USA, the only other campaigns that partnered with a health insurance provider were in France. The European Centre for Disease Control contributed to the bulk of projects which we tagged as intergovernmental organizations.

Professional societies, particularly those with a strong tie to AMR, were heavily involved in the creation and provision of resources for HCWs. Three examples are the British Society for Antimicrobial Chemotherapy, which has been involved in the creation of resource websites (Initiative #48), coordination of a MOOC (Initiative #90), and provision of funding for AMR education; the European Society of Clinical Microbiology and Infectious Diseases, which runs a study group on antimicrobial resistance, develops and coordinates courses, and carries out collaborative academic research; and the Infection Control Africa Network which organized a free distance learning course for HCWs in Africa in 2014. Many professional bodies with broader mandates, for example, medical associations and pediatric societies, have partnered with others to support larger projects, but were rarely the lead organization in the projects that we identified. For example, the NPSMedicineWise project (Initiative #82) in Australia engaged the Australian College of Nursing, Australian Association of Consultant Pharmacy, the Australian College of Rural & Remote Medicine, and the Australian Nursing and Midwifery Federation and many others alongside government bodies to produce resources and course content for the website.

University-led projects were typically related to online or in-person courses or to resource websites. Many of these sites provide lecture slides, or recorded video lectures (e.g. Initiative #16), as well as information on guidelines and policies (e.g. Initiative #35). Other types of organization were engaged in a wide variety of projects. Hospitals and healthcare centers, for example, engaged with umbrella programs, courses and workshops, intervention studies, and resource guides.

**Fig. 3:**
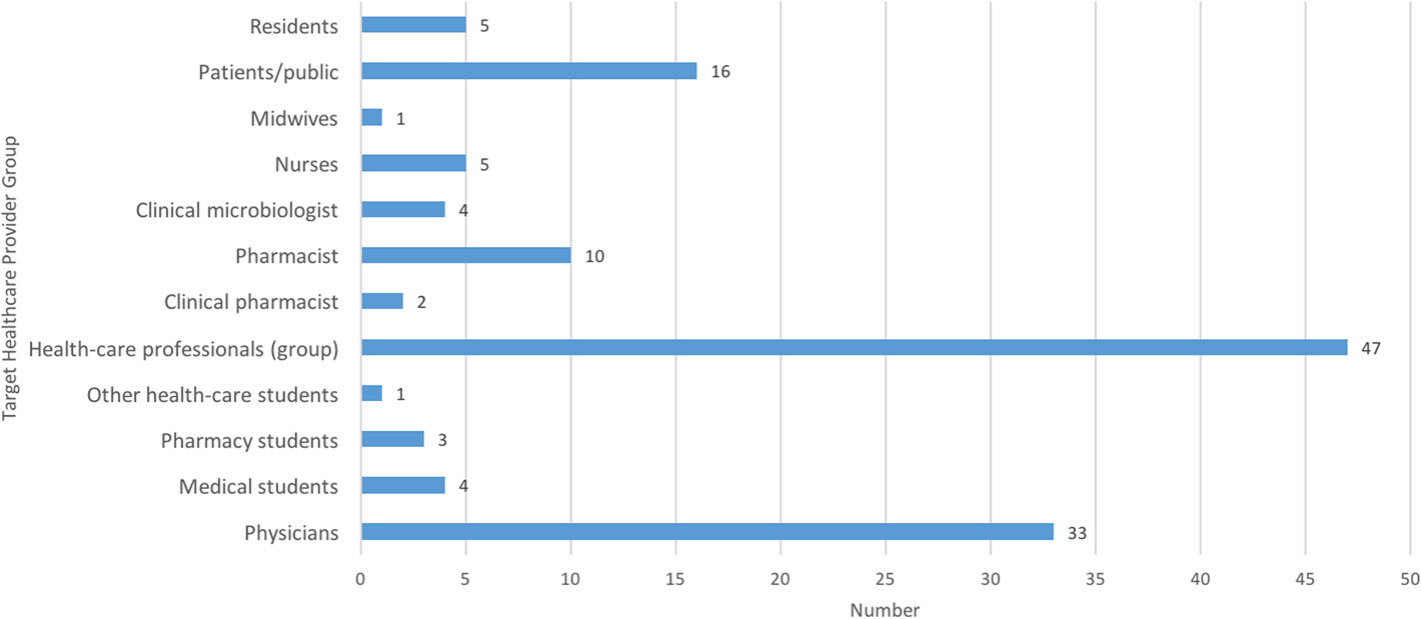
Number of identified AMR education programs by target healthcare worker group

Most programs targeted HCWs as a broadly defined group. Figure 3 shows the target audiences for the 94 programs identified. Several programs also included components aimed at patients and the public. We found 33 programs aimed at physicians, including a small number that targeted specialists such as infectious disease physicians and pediatricians. Fewer programs specifically targeted nurses, pharmacists, and midwives, though these groups were targeted in the more general HCW programs. A small number of predominantly university-based programs targeted medical or pharmacy students. Four programs, mostly courses and workshops, targeted clinical microbiologists alongside other HCWs.

**Fig. 4:**
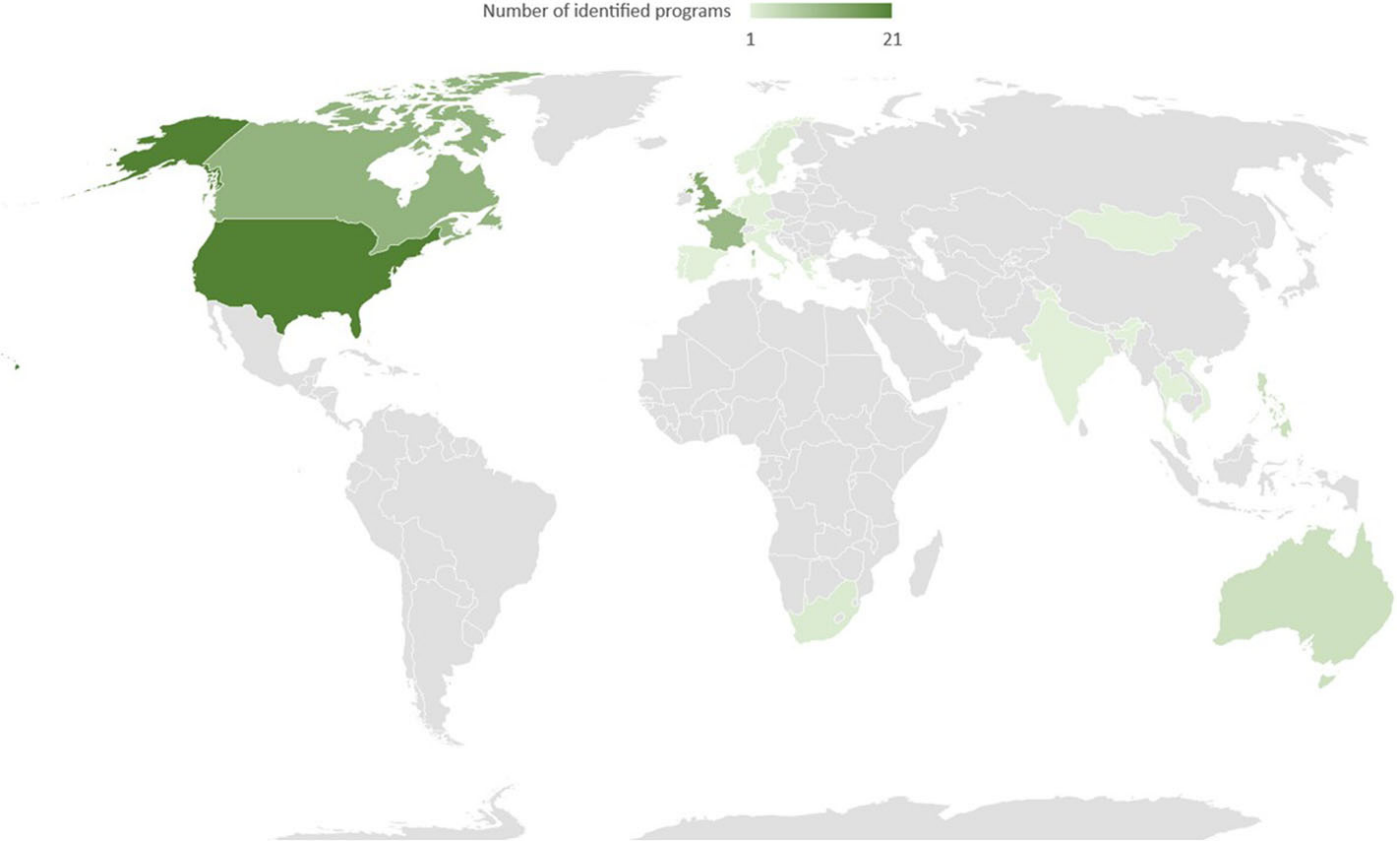
Map of AMR education programs by region. Map was generated using Microsoft Excel and Bing

We identified programs from around the world, though, as expected, the programs were unequally distributed between countries and regions, with a larger share of the programs from North America and Europe. Figure 4 shows a map of the number of programs identified from each region. Many of the large-scale AMR/AMS educational campaigns took place in wealthy American and European countries (e.g. Initiatives #15 and #59), whereas many of the programs in Africa and Asia were workshop or course based (e.g. Initiatives #1, #78, #83). The interconnected nature of some programs made them more easily producible on the part of the organizations and more easily identifiable on our part as researchers. This strategy facilitated the growth of programming in specific regions, and has made online information about AMR/AMS widely available to HCWs and the public. For instance, state-specific AMR educational initiatives, such as “Get Smart Colorado: Use Antibiotics Wisely” (Initiative #17), appeared to tailor resources or share/endorse existing resources from the comprehensive national “Get Smart” program (Initiative #15) produced by the CDC in the USA.

## Discussion

Our environmental scan shows that many organizations are working to develop and share course content and educational resources for HCW training and continuing education around the world. We identified several types of resources, from traditional in-person courses to workshops and conferences, to MOOCs, to online campaign materials, to handbooks, and to workbooks. Governments, hospitals, and professional societies appear to be driving action on this front, and these three types of organization have successfully built partnerships with others including non-profits, insurance companies, and WHO regional offices.

This level of collaborative work is promising; however, we have identified opportunities for action on several fronts. First, there is an opportunity for the development of better resources and training opportunities for students in the healthcare professions. Early engagement can provide a strong foundation of AMR knowledge that students draw upon as they progress through their careers as prescribers and patient educators. Second, it appears that training for current HCWs is probably under-recognized by professional development and continuing education programs. HCWs need to have their AMR learning efforts formally recognized, as they may lack incentives to engage in substantial additional training on a purely volunteer basis. Below we discuss how addressing these two elements of training, both pre-service and continuing education, can improve the content, delivery, uptake, and scale-up of AMR/AMS education, and identify programs that are interesting models for development of further resources.

### Pre-service student engagement

We identified a small number resources for students in healthcare disciplines, though research suggests that medical students in Europe and the USA have a desire for increasing training on AMR [11, 12]. Researchers from the ESCMID Study Group for Antibiotic Policies have suggested that undergraduate training should be focused on principles of AMS and building good prescribing habits [12]. Resources on these topics are available, as the MOOCs we identified often included lectures, videos, and case studies on these themes. Students, however, are unlikely to have the time to pursue these courses outside their educational program. Educational research also suggests that training at the undergraduate level should ideally be delivered through active learning sessions which allow students to integrate knowledge with practice [12, 13]. We note that the small number of programs specifically targeting students have embraced opportunities for active student participation and interaction. To support their curriculum for medical students (Initiative #16), Wake Forest University designed series of small-group case studies with facilitator guides to allow group discussions of AMR issues. In Canada, the Do Bugs Need Drugs? Program (Initiative #14) has engaged students from medicine, pharmacy, nursing, and dentistry in delivering educational messages on AMR to students in elementary school. Canadian medical students have also been active in developing their own opportunities for AMR/AMS learning. In 2014, students at the University of Toronto created the Students for Antimicrobial Stewardship Society, which has now expanded to include students in dentistry and pharmacy and at other medical schools. The students have launched an annual awareness campaign on AMR, and organized educational lectures and interprofessional panels on stewardship to provide students with opportunities to ask questions (Initiative #37). Given the small number of programs that engage with students, there are undoubtedly more opportunities to develop resources for students. These might include the creation of educational curricula and support resources including videos and case studies, or engagement of students and student societies in broader educational programs.

### Continuing education

We found that many excellent courses already exist, and several are available online at low or no cost. As content is already available, improving roll-out is a matter of ensuring that these courses can run repeatedly; preparing a course for a single session is likely a waste of resources. Another way to improve roll-out is to facilitate translation of course materials to other languages. Consulted experts noted that translating available content, such as a MOOC, from English into other languages is a slow and difficult process. Beyond the translation of lecture slides, there is a need to obtain other resources, such as academic articles and video content, in the foreign language. Creation of a database that brings together resources on AMR/AMS in other languages would facilitate the adaptation of courses between languages.

Improving uptake of existing educational opportunities is also key. We note that among the resources identified, very few are accredited as continuing professional education. This is probably a major oversight—and opportunity—as evidence suggests that HCWs are not receiving enough training on AMR/AMS [14]. HCWs would likely be incentivized to participate if the courses were accredited as CME, as HCWs in many countries must undertake several hours of CME per year. Experts that we consulted expressed a desire for courses qualifying as CME, but found the accreditation process challenging and slow. To date, most courses have been developed by academic societies and there is an opportunity here for greater collaboration between academic societies and professional societies. Academic societies have knowledge and resources at their disposal and, by partnering with professional societies such as pediatric societies or nurses associations, could adapt their existing programs to meet the specific training needs of these healthcare specializations and provide training to a larger number of HCWs. Regulators and professional councils should be engaged in devising strategies to facilitate the uptake and scale-up of AMR-related pre-service education and in-service training initiatives.

### Other mechanisms of engagement

Many of the programs that we identified made their content freely available online. In addition to courses, we found lecture slides, video lectures, case studies, facilitator guides, handbooks, posters, and other campaign materials online. The presence of these many high-quality resources online suggests that it is not necessary for new educational campaigns and courses to reinvent the wheel; a large amount of information is already available. However, much of this content is scattered across the web. It would be very useful to develop a resource site or database of good AMR content. Collecting this content would allow educators to build off of existing educational materials and focus their efforts on developing course content specific to the local context. The ReAct Toolbox (Initiative #94) is an excellent model, providing basic information on several aspects of AMR/AMS and links to other online resources that are relevant to both LMICs and high-income countries. The main limitation of the site, and many of the other educational resources that we have identified, is that many resources are only available in English.

### Strengths and limitations of this study

This project is the first attempt that we know of to map educational resources for HCWs around the world. We contacted more than 50 experts and identified educational resources and projects in all WHO regions. By identifying and classifying programs, we can see the breadth of resources already available for training HCWs on AMR/AMS.

As we relied extensively on web searching in addition to expert advice, it is likely that we identified more of some types of resource than others. Therefore, the quantity of identified programs of this type should not be interpreted as proof that these resources are the most common program type. We paired our web searching with expert input to identify those other resources that are more difficult to identify online. We recognize that some web resources, such as guidelines, are more likely to endure on the internet than others, such as advertisements for courses and conferences. We included mobile apps specific to antibiotic resistance if they were identified by experts or identified in web searches. However, it was beyond the scope of the study to search within existing medical apps to locate AMR/AMS content; this has been done elsewhere by Goff et al. [15].

We conducted our web and academic searching in English and relied upon on expert contacts to provide information on educational programs in other languages. We identified and reviewed some educational programs where the detailed information online was in French, but did not translate articles from other languages, and therefore likely missed other educational resources posted online that are only available in other languages.

Finally, we note that longevity of resources on the internet is an issue. We found many broken links, where program information could no longer be obtained. We identified many courses through course advertisements posted to the web; however, the limitations of our search strategy make it likely that we mainly identified recent courses. Further, for most courses, we cannot comment as to whether the course content has been archived on the web. While much of the content from a larger course, such as a MOOC, is likely to remain on the web, it is unlikely that the course content of shorter, in-person courses will be retained in the same way. Resource sites must monitor content to ensure it remains up to date. To facilitate the sharing of resources between countries, it will also be important to find a mechanism such that content does not become inaccessible after removal from the website.

## Conclusions

In mapping the available educational programs on AMR/AMS for HCWs, we have identified 94 existing programs and resources, and several opportunities for improvement and expansion on the existing resources. Many high-quality educational campaigns already exist and there are many opportunities to adapt these programs to meet educational needs in other contexts. But gaps exist, particularly when it comes to resources for healthcare students, and provision of accredited training for current HCWs. National and international stakeholders should advocate for increased accreditation, the creation of a series of competencies for undergraduate curricula, and support resource sharing platforms. Action on these fronts would support both the goals laid out in many national AMR action plans and the desires of many HCWs to ensure that all HCWs receive sufficient training on AMR and AMS.

## Abbreviations

AMR: Antimicrobial resistance
AMS: Antimicrobial stewardship
BSAC: British Society for Antimicrobial Chemotherapy
CDC: Centers for Disease Control and Prevention
HCWs: Healthcare workers
IPC: Infection prevention and control
MOOC: Massive open online course
USA: United States of America
